# Safety and efficacy of elapegademase in patients with adenosine deaminase deficiency: A multicenter, open‐label, single‐arm, phase 3, and postmarketing clinical study

**DOI:** 10.1002/iid3.917

**Published:** 2023-07-25

**Authors:** Masafumi Onodera, Toru Uchiyama, Tadashi Ariga, Masafumi Yamada, Takako Miyamura, Hironori Arizono, Tomohiro Morio

**Affiliations:** ^1^ Division of Immunology National Center for Child Health and Development Tokyo Japan; ^2^ Department of Pediatrics, Faculty of Medicine, Graduate School of Medicine Hokkaido University Sapporo Japan; ^3^ Department of Food and Human Wellness Rakuno Gakuen University Ebetsu Japan; ^4^ Department of Pediatrics Osaka University Graduate School of Medicine Suita Japan; ^5^ Pharmaceutical Development & Production Division Teijin Pharma Limited Tokyo Japan; ^6^ Department of Pediatrics and Developmental Biology Tokyo Medical and Dental University Tokyo Japan

**Keywords:** adenosine deaminase deficiency, elapegademase, enzyme replacement therapy, immunologic deficiency syndromes

## Abstract

**Introduction:**

Adenosine deaminase (ADA) deficiency is an ultrarare inherited purine metabolism disorder characterized by severe combined immunodeficiency. Elapegademase‐lvlr is a new pegylated recombinant bovine ADA used in enzyme‐replacement therapy (ERT) for ADA deficiency. Therefore, replacement with the new drug may eliminate the infectious risks associated with the currently used bovine intestinal‐derived product, pegademase.

**Methods:**

We conducted a multicenter, single‐arm, open‐label, phase 3, and postmarketing clinical study of elapegademase for patients with ADA deficiency. The following biochemical markers were monitored to determine an appropriate dose of elapegademase: the trough deoxyadenosine nucleotide (dAXP) level ≤0.02 μmol/mL in erythrocytes or whole blood and the trough serum ADA activity ≥1100 U/L (equivalent to plasma levels ≥15 μmol/h/mL) indicated sufficient enzyme activity and detoxification as efficacy endpoints and monitored adverse events during the study as safety endpoints.

**Results:**

A total of four patients (aged 0–25 years) were enrolled. One infant patient died of pneumonia caused by cytomegalovirus infection whereas the other three completed the study and have been observed in the study period over 3 years. The infant patient had received elapegademase at 0.4 mg/kg/week until decease and the others received elapegademase at maximum doses of 0.3 mg/kg/week for 164–169 weeks. As a result, all four patients achieved undetectable levels of dAXPs together with sufficient enzyme activity, increased T and B cell numbers, and slightly elevated and maintained IgM and IgA immunoglobulin levels. Serious adverse events occurred in three patients, all of which were assessed as unrelated to elapegademase.

**Conclusions:**

This study showed that elapegademase had comparable safety and efficacy to pegademase as ERT for ADA deficiency by demonstrating stable maintenance of sufficient ADA activity and lowering dAXP to undetectable levels, while no drug‐related adverse events were reported (Trial registration: JapicCTI‐163204).

## Graphical abstract

Time course changes in the trough dAXP concentrations of erythrocytes and whole blood.

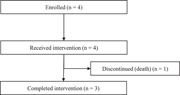

## 1 INTRODUCTION

An inherited deficiency of adenosine deaminase (ADA) is an ultrarare metabolic disorder (one in 0.2–1 million newborns) resulting in the accumulation of toxic purine metabolites, especially deoxyadenosine triphosphate (dATP).^1^, ^2^ The accumulated toxic metabolites have deleterious damage on the development of immature T cells in the thymus as a cause of the development of severe combined immunodeficiency (SCID),^2^, ^3^, ^4^, ^5^, ^6^ which is characterized by severe opportunistic infections, diarrhea, and failure to thrive from birth if not treated properly within the first year of life.^1^, ^2^ The current curative treatments for the disease are hematopoietic cell transplantation (HCT)^1^, ^2^ and hematopoietic stem cell gene therapy (HSC‐GT).^7^, ^8^ However, the former requires a suitable donor such as an HLA‐matched one, especially an HLA‐matched sibling donor in the case of ADA deficiency, and the latter is only feasible in very limited counties.^9^, ^10^, ^11^

On the other hand, since ADA deficiency is also classified as an inherited metabolic disorder, enzyme replacement therapy (ERT) is available as an effective therapeutic option for this disease. ERT was started in the United States in 1990 with pegylated bovine ADA, pegamedamse, and has been used in the EU since the 2000s. Since ERT can be relatively easily introduced to patients who require improvement of their pre‐HCT status and can be used as a long‐term (or lifelong) treatment option for patients who are ineligible or unresponsive to the curative treatments,^8^, ^12^, ^13^, ^14^, ^15^, ^16^ it is recommended as the first‐line treatment for ADA deficiency by the 2019 guideline.^17^ However, it has been reported that long‐term use of pegademase gradually causes a decline in immune function and up to 20% of patients become treatment‐resistant. As such, it is currently considered a bridge treatment to curative therapies such as HCT or HSC‐GT. In addition, pegademase is a product of bovine intestinal origin and therefore it has theoretical infectious risks such as pathogen infection and transmissible spongiform encephalopathies (TSEs). Although pegademase has been used in over 150 patients worldwide,^3^ a new recombinant version of bovine ADA conjugated to polyethylenglycol elapegademase‐lvlr has recently been developed and approved in the United States in 2018 based on the results of a 21‐week switch study protocol from pegademase for ADA‐deficient patients.^18^

On the other hand, ERT with pegademase is not approved in Japan, and one patient with ADA deficiency has been treated so far under a publicly funded research program. Thus, we conducted a phase 3 study to evaluate the safety and efficacy of elapegademase in Japanese patients with ADA deficiency. This report presents data from the study composed of an evaluation phase and a continuous administration phase, the latter of which includes a postmarketing clinical study. Since very few prospective clinical studies have been published so far, our study provided limited, but significant information on elapegademase for ADA deficiency.

## 2 MATERIALS AND METHODS

### 2.1 Study design and patients

The phase 3 clinical trial was conducted from March 2, 2016, to March 26, 2019, followed by the postmarketing clinical study from March 27 to June 5, 2019. The trial was conducted in accordance with the ethical principles of the Declaration of Helsinki and the Good Clinical Practice guidelines. All patients or their guardians provided their written informed consent. The study protocol was approved by the institutional review board at each center (Hokkaido University Hospital, Sapporo, Japan; National Center for Child Health and Development, Tokyo, Japan; Osaka University Hospital, Osaka, Japan). The trial registration number was JapicCTI‐163204. This manuscript adhered to the Consolidated Standards of Reporting Trials statement.

This open‐label, uncontrolled clinical study of elapegademase was conducted at three centers in Japan. The study consisted of an evaluation phase (including a 5‐week dose adjustment period and a 16‐week dose maintenance period) and a continuous administration phase wherein continued administration until the completion of the study was planned. Since ADA deficiency is a serious disease, the phase 3 study was extended to continuously provide patients with elapegademase beyond the day of marketing approval in Japan (granted in March 2019 based on data from the evaluation phase).

To be included in the study, patients were required to have been genetically diagnosed with ADA deficiency or diagnosed based on clinical symptoms and ADA activity by the investigator, and judged by the investigator to require ERT. Patients with severe thrombocytopenia (platelet count < 50 × 10^9^ cells/L) and those who were pregnant or lactating were excluded. The use of vidarabine, nelarabine, and pentostatin as ADA inhibitors were prohibited from the time of informed consent to the end of the continuous administration phase. The use of the following drugs and therapies was prohibited from the time of informed consent to the end of the evaluation phase: pegademase, HCT, HSC‐GT, and other investigational products.

### 2.2 Treatment and procedures

The study drug was provided as a solution for injection containing 2.4 mg (1.6 mg/mL) of elapegademase (a polyethylene glycol‐modified recombinant bovine ADA analog; Leadiant Biosciences) in a 1.5‐mL vial. It was injected intramuscularly once a week. The doses ranged from 0.067 to 0.2 mg/kg and increased or decreased by 0.033 mg/kg at specified times.

#### 2.2.1 Evaluation phase; dose adjustment period

For the patients without treatment of pegademase before informed consent, elapegademase with 0.1 mg/kg of the dose was injected at the 1st administration and 0.133 mg/kg at the 2nd and 3rd administration, respectively. For the patients with treatment of pegademase before informed consent, based on the latest doses of pegademase, the dose of elapegademase from the 1st to 3rd administration was determined, not exceeding the dose calculated from the conversion formula shown below, out of 0.067, 0.1, 0.133, 0.167, or 0.2 mg/kg.

[Conversionformula]:Dose of elapegademase (mg/kg)=Dose of pegademase (U/kg)×1mgelapegademase/150Upegademase.

The doses of the 4th and 5th administration were modified if any of the dose modification criteria were observed.

#### 2.2.2 Evaluation phase; dose maintenance period

The same dose of the 5th administration during the dose adjustment period was continuously injected at the 6th administration if the dose modification was not needed. After the 9th, 13th, or 17th administration, if any of the dose modification criteria were observed, the next dose was modified.

#### 2.2.3 Criteria of dose modification in the evaluation phase

Criteria of dose modification during the dose adjustment period were set as follows: (1) erythrocyte or blood deoxyadenosine nucleotide (dAXP) concentration, >0.02 μmol/mL, (2) serum ADA activity level, <1100 U/L, (3) the onset, unchanged status, worsening of clinical symptoms or variations of the values and abnormal values of the clinical laboratory data of immune functions, hepatic functions, and so forth, and (4) safety concerns.

Criteria of dose modification during the dose maintenance period were set as follows: items (1) and (2) defined above were observed for the last two measurements, and the other items (3) and (4) were the same as during the dose adjustment period.

#### 2.2.4 Continuous administration phase

Based on the information obtained in the evaluation phase, we determined the maintaining doses and also the necessity of the dose modification after every four‐time administration in principle, considering the efficacy and safety.

Moreover, during the dose maintenance period and in the continuous administration phase, when the need for increased dose is determined considering the clinical symptoms of the patients and also erythrocyte dAXP concentration of ≤0.02 μmol/mL (blood dAXP could be used if an erythrocyte result was unavailable) and serum ADA activity of ≥1100 U/L were not achieved, it was accepted to inject elapegademase at a dose of 0.233 mg/kg or more.

### 2.3 Endpoints and assessments

The primary endpoint was not defined in this study. The efficacy endpoints were trough values of erythrocyte dAXP concentration of ≤0.02 μmol/mL, blood dAXP concentration of ≤0.02 μmol/mL, serum ADA activity level of ≥1100 U/L, plasma ADA activity level of ≥15 µmol/h/mL, and immune functions including lymphocyte, lymphocyte subset, and immunoglobulin. dAXP, a series of phosphorylated products of an ADA substrate, deoxyadenosine, was the cause of inducing various kinds of deficits in patients with ADA deficiency and has been considered one of the important markers for efficacy evaluation. ADA activity was determined to confirm the enzyme replaced by elapegademase administration. Both of them were also set to determine the dose of elapegademase. Plasma and serum ADA activity was determined by the conversion ratio from adenosine to inosine and the decrease in NADPH, respectively. Erythrocyte and blood dAXP were determined by UV‐HPLC and LC‐MS/MS methods, respectively. The key safety endpoint was adverse events (AEs) and adverse drug reactions (ADRs).

The dAXP concentration and ADA activity level were measured at the following time points: at screening, the 2nd, 3rd, 4th, and 5th administration of the dose adjustment period, the 6th, 7th, 10th, 11th, 14th, 15th, 18th, and 19th administration of the dose maintenance period (when the dose is modified at the 6th, 10th, 14th, or 18th administration of the dose maintenance period, additional measurement was performed at 8th, 12th, 16th, or 20th administration), the end of the evaluation phase, the end of the evaluation phase, the *z*th administration (“*z*” is equal to 16n + 22) of the continuous administration phase, and the end of the continuous administration phase or discontinuation. The immune functions were measured at the following time points: at screening, the 3rd administration of the dose adjustment period, the 6th, 10th, 14th, and 18th administration of the dose maintenance period, the end of the evaluation phase, the *z*th administration (when *z* is equal to 16n + 22) of the continuous administration phase, and the end of the continuous administration phase or discontinuation. The AEs were observed throughout the study period. AEs were defined as any undesirable or unintended signs, symptoms, diseases, abnormal variation of values of clinical laboratory tests (hematology, blood biochemistry, and urinalysis), or vital signs that occurred in the subject from the start of the study drug administration to the end of the study. In addition, when the events (signs, symptoms, or diseases) observed before administration of the study drug became worse after the start of the study drug administration compared with those observed before administration, the events were judged as AEs. AEs unable to rule out the causal relationship to the study drug was handled as “an ADRs.” Criteria for the severity were as follows: Mild; Treatment is not required. The patient is able to continue the study. Moderate; Treatment is required. The study drug is reduced or interrupted. Severe; Treatment is required. The study is discontinued.

### 2.4 Statistical analysis

The frequency of ADA deficiency overseas is 1 out of 0.2–1 million newborn babies. In 2014, the number of patients was estimated to be four in Japan. Out of them, two patients needed treatment with ERT, therefore, considering the feasibility, the number of patients in this study was set to two. More patients could be enrolled after two patients were enrolled. The efficacy (and safety) analysis population included all enrolled patients who received at least one dose of the study drug and at least one postbaseline efficacy (and safety) assessment. For the efficacy analysis, individual raw data were graphed by the measurement time points; moreover, patient characteristics and treatment, and a summary of AEs and ADRs were listed. The number and proportion of subjects who experienced AEs or ADRs and the number of AEs or ADRs were aggregated using the MedDRA Japanese version 19.1. SAS version 9.3 was used for all analyses.

## 3 RESULTS

### 3.1 Patient enrollment

**Figure 1:**
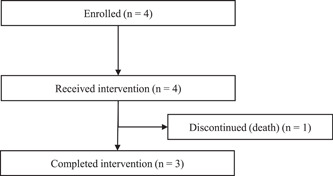
Study flowchart.

Four patients numbered Patient‐1, ‐2, ‐3, and ‐4 (Pt‐1, ‐2, ‐3, and ‐4) were enrolled in the study and received the study drug (Figure 1). Although all the subjects had been observed in the dose adjustment period, one patient, Pt‐4, was excluded from the study due to death by pneumonia caused by cytomegalovirus (CMV) infection in the dose maintenance period and the other three completed the study. The study performed the efficacy and safety analysis using the clinical data of these four subjects in the study. The breakdown of the subjects was two males, two females, two under 15 years old, and two over 15 years (range, 0–25 years old) as shown in Table 1. Pt‐2 and Pt‐3 had been treated with pegademase within 60 days before informed consent, and Pt‐1 and Pt‐2 had received HSC‐GT as described.^10^ Pt‐4 was given a high dose of the study drug from the first administration to avoid fatal situations, which was noted as a protocol deviation and used as a reference to set the maximum dose of 0.2 mg/kg twice a week for severely ill patients in the protocol amended in March 2018.

**Table 1:** Patient characteristics and treatment.

Patient	Patient‐1	Patient‐2	Patient‐3	Patient‐4^a^
Age	≥15 years	≥15 years	Infant	Infant
Sex	Male	Female	Female	Male
Use of pegademase^b^	No	Yes (32 U/kg)	Yes (28 U/kg)	No
Prior curative treatment for ADA deficiency^c^	Yes	Yes	No	No
Dose of study drug,^d^ mg/kg/week				
1st dose, dose adjustment period	0.1	0.2	0.167	0.4
6th dose, dose maintenance period	0.167	0.2	0.2	0.4
22nd dose, continuous administration phase	0.167	0.267	0.233	Not applicable
Last dose, continuous administration phase	0.167	0.3	0.233	Not applicable
At the time of dose modification based on findings/symptoms (increased dose, mg/kg/week)	4th dose in dose adjustment period (0.167)	10th dose in dose maintenance period (0.233); 18th dose in dose maintenance period (0.267); 38th dose in continuous administration phase (0.3)	4th dose in dose adjustment period (0.2); 10th dose in dose maintenance period (0.233)	Not applicable
Number of doses				
Evaluation phase	21	21	21	16
Continuous administration phase (postmarketing clinical study)	143 (9)	148 (10)	144 (9)	Not applicable
Throughout the study^e^	164	169	165	16

^a^ A severely ill infant continued to be treated with a high dose (0.2 mg/kg twice weekly) until death.

^b^ Treatment with pegademase within 60 days before informed consent.

^c^ Hematopoietic stem cell transplantation or gene therapy.

^d^ Patients not treated with pegademase before study entry were treated at 0.1 mg/kg for the 1st dose and 0.133 mg/kg for the 2nd and 3rd doses. Following the protocol amendment (March 2018), this regimen was changed to 0.2 mg/kg for the 1st through 5th doses.

^e^ The number of doses administered between the start and end of the study (evaluation phase + continuous administration phase).

### 3.2 Clinical course and efficacy

**Figure 2:**
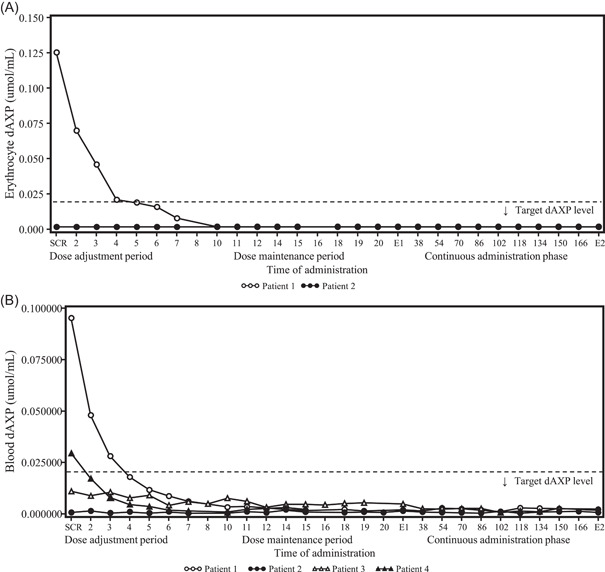
Time course changes in the trough dAXP concentrations. Erythrocyte dAXP (A) and blood dAXP (B) at each time point. The target erythrocyte (or blood) dAXP level was set at ≤0.02 µmol/mL. Time of administration indicates the following time points: the 2nd to 5th doses in the dose adjustment period; the 6th to 20th doses in the dose maintenance period; and the 38th to 166th doses in the continuous administration phase. dAXP, deoxyadenosine nucleotide; E1, at the end of the evaluation phase; E2, at the end of the continuous administration phase; SCR, screening.

**Figure 3:**
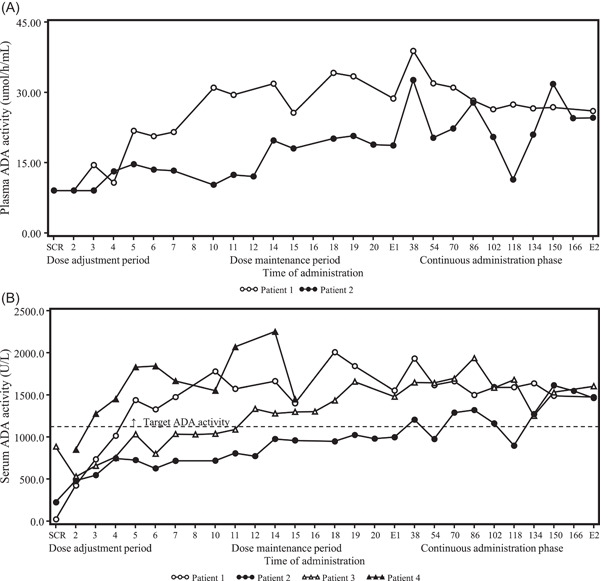
Time course changes in the trough ADA activity. Plasma ADA activity (A) and serum ADA activity (B) at each time point. The target serum ADA activity level was set to at least 1100 U/L (equivalent to at least 15 µmol/h/mL in plasma). Time of administration indicates the following time points: the 2nd to 5th doses in the dose adjustment period; the 6th to 20th doses in the dose maintenance period; and the 38th to 166th doses in the continuous administration phase. ADA, adenosine deaminase; E1, at the end of the evaluation phase; E2, at the end of the continuous administration phase; SCR, screening.

**Figure 4:**
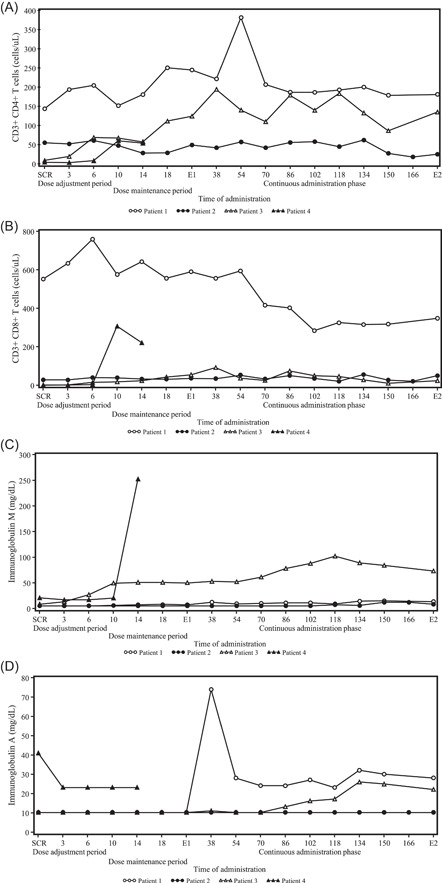
Time course changes in the immune function: T cell count. CD3^+^ CD4^+^ T cells (A) and CD3^+^ CD8^+^ T cells (B) at each time point. Time of administration indicates the following time points: the 2nd to 5th doses in the dose adjustment period; the 6th to 20th doses in the dose maintenance period; and the 38th to 166th doses in the continuous administration phase. Time course changes in the immune function: immunoglobulin. Immunoglobulin M (C) and immunoglobulin A (D) at each time point. Time of administration indicates the following time points: the 2nd to 5th doses in the dose adjustment period; the 6th to 20th doses in the dose maintenance period; and the 38th to 166th doses in the continuous administration phase. E1, at the end of the evaluation phase; E2, at the end of the continuous administration phase; SCR, screening.

Dosage and administration records are summarized in the supplementary table. Changes in trough erythrocyte or blood dAXP concentrations and trough plasma or serum ADA enzyme activity levels over time are shown in Figures 2 and 3, respectively, and CD3^+^ CD4^+^ and CD3^+^ CD8^+^ T lymphocyte count and IgM and IgA levels as immune functions are shown in Figure 4. The dose was adjusted based on levels of trough dAXP concentrations and ADA enzyme activity.

#### 3.2.1 Patient‐1

Pt‐1 was a Japanese male over 15 years old who had received T‐cell‐directed gene therapy at 5 years old^9^ and HCT‐GT at 13 years old^10^ and then has been treated with prophylactic antibiotics and gamma globulin replacement therapy. He did not receive pegademase within 60 days before informed consent. At the screening, the dAXP concentrations in his erythrocytes and blood were 0.125 and 0.0952 μmol/mL and ADA enzyme activity in his plasma and serum were <9.0 μmol/h/mL and <1100 U/L, respectively. The dose started at 0.1 mg/kg and increased to 0.133 mg/kg at the 2nd and 3rd administrations resulting in a decrease of the trough dAXP concentrations in his erythrocytes and blood to 0.070 and 0.0477 μmol/mL at 2nd administration and 0.046 and 0.0279 μmol/mL at 3rd administration, respectively. In contrast, the trough levels of ADA enzyme activity increased to 421.1 U/L in his serum at 2nd administration and 14.51 μmol/h/mL and 733.5 U/L in both plasma and serum at the 3rd administration. Since more dose was needed to satisfy the dose modification criteria, the dose was increased to 0.167 mg/kg at the 4th administration and the dAXP concentration was gradually decreasing in both his erythrocytes and whole blood and reached the target level ≤0.02 μmol/mL at the 5th administration. Since the trough ADA enzyme activity in his plasma and serum also reached ≥15 μmol/h/mL and ≥1100 U/L of target values at the 5th administration, the subsequent dose was fixed to 0.167 mg/kg in the dose maintenance period and the continuous administration phase, during which the values of dAXP and ADA enzyme activity remained stable and within the target value.

Since Pt‐1 had already received T‐cell‐directed gene therapy and HSC‐GT previously, he had a certain number of peripheral lymphocytes including CD3^+^ CD4^+^ and CD3^+^ CD8^+^ T cells at the screening. After starting ERT with elapegademase, his CD3^+^ CD4^+^ T cells increased gradually from 143 cells/μL at the screening to 244 cells/μL at the end of the evaluation phase. After that, it remained stable between 178 and 206 cells/μL during the continuous administration phase with a transient peak of 381 cells/μL at the 54th administration. On the other hand, his CD3^+^ CD8^+^ T cells decreased gradually from 552 cells/μL at the screening during the whole study to 348 cells/μL at the end of the study. Regarding the immune globulin levels, while IgM levels increased slightly from less than 5 mg/dL at the screening to 13 mg/dL at the end of the study, a significant increase in IgA levels was observed from less than 10 mg/dL at the screening to 28 mg/dL at the end of the study with a transient peak of 74 mg/dL at 38th administration.

#### 3.2.2 Patient‐2

Pt‐2 was a Japanese female over 15 years old. Although she had received HSC‐GT at 4 years old^10^ and had been treated with prophylactic antibiotics and gamma globulin replacement therapy, she restarted ERT with pegademase at 11 years old due to poor weight gain with intractable pneumonia and liver dysfunction and had received it for 5 years until the enrollment of the study. The initial dose was calculated with the conversion formula between pegademase and elapegademase and decided to be 0.2 mg/kg equivalent to the dose of pegademase used. While the dAXP concentrations in her erythrocytes and whole blood had kept the target level (≤0.02 μmol/mL) during the whole study, the ADA enzyme activity in her plasma and serum increased gradually from <9.0 μmol/h/mL and 223.7 U/L at the screening to 14.68 μmol/h/mL and 724.0 U/L at the 5th administration, although they were still below the target values of ≥15 µmol/h/mL or ≥1100 U/L, respectively. Then, the dose increased to 0.233 mg/kg at the 10th administration and maintained until the 17th administration in the dose maintenance period. As a result, the plasma ADA enzyme activity exceeded 15 μmol/h/mL after the 14th administration. To expect a further increase in serum ADA enzyme activity, the dose increased to 0.267 mg/kg at the 18th administration and finally to 0.3 mg/kg at the 38th administration. With some increase/decrease of the serum ADA enzyme activity, the trough levels were around or above 1100 U/L after the 38th administration.

Because she had already been treated with pegademase, no significant change of the CD3^+^ CD4^+^ and CD3^+^ CD8^+^ T cell counts was observed during the whole study, and the serum immunoglobulin levels such as IgM and IgA levels changed around 10 mg/dL from the screening to the end of the study.

#### 3.2.3 Patient‐3

Pt‐3 was a Japanese female infant diagnosed with ADA deficiency at 2 months, suffering from poor weight gain with severe lymphopenia, and subsequently treated with pegademase for 1 month before study entry. To reduce the total volume of blood collection, the blood dAXP concentrations and serum ADA enzyme activity were only measured and they were under 0.02 μmol/mL and 884 U/L at the screening, respectively. The study drug with 0.167 mg/kg of the dose calculated by the conversion formula was injected into the patient until the 3rd administration and then increased to 0.2 mg/kg due to satisfying the criteria at the 4th administration and continued to the end of the dose adjustment period. While the trough blood dAXP concentration remained under 0.02 μmol/mL of target value during the whole study, her serum ADA enzyme activity increased by a degree and reached 1100 U/L at the 12th administration of the dose maintenance period and maintained this value until the end of the study.

Although the number of CD3^+^ CD8^+^ T lymphocytes showed no significant increase, CD3^+^ CD4^+^ T lymphocytes increased from 8 cells/μL at the screening to around 150 with 193 cells/μL at peak and the value of IgM and IgA in her serum also increased from 8 and <10 mg/dL at the screening to around 70 and 20 mg/dL in the continuous administration phase, respectively, although the increase of IgA level was observed 10 months later than that of IgM.

#### 3.2.4 Patient‐4

Pt‐4 was a Japanese male infant diagnosed with ADA deficiency at 4 months of age who suffered from severe pneumonia caused by CMV infection and enrolled to receive the treatment with the study drug. The blood dAXP concentration was 0.0293 μmol/mL, and the serum ADA enzyme activity was not determined to reduce the amount of blood collection at the screening. Because of his extremely critical condition, the study drug started with 0.2 mg/kg twice a week of dose from the beginning as deviance, which was conducted as an in‐house approval of the sponsor with the understanding of the Pharmaceuticals and Medical Devices Agency (PMDA). With the high dose of the study drug, the dAXP in his blood declined rapidly and reached the target level of below 0.02 μmol/mL at the 2nd administration and ADA enzyme activity in his serum also increased rapidly and reached ≥1100 U/L at the 3rd administration, respectively. Although such a high dose of the study drug maintained undetectable levels of dAXP and over 1100 U/L of ADA enzyme activity during hospitalization, his condition was getting worse despite multiple drug treatments of antibiotics and anti‐CMV drugs and died of pneumonia caused by CMV infection after the 16th administration.

The number of CD3^+^ CD4^+^ T lymphocytes increased from 3 cells/μL at the screening to 53 cells/μL at the 14th administration. Of interest was the rapid increase of CD3^+^ CD8^+^ T lymphocytes up to 221 cells/μL at the 14th administration, which was not observed in other cases and likely showed the proliferation of cytotoxic T lymphocytes against CMV. Although serum IgA levels decreased to below the detection limit (less than 23 mg/dL) from 41 mg/dL at the screening, the IgM levels suddenly increased from 21 mg/dL at the screening to 250 mg/dL at the 14th administration, suggesting the existence of specific IgM against CMV.

### 3.3 Safety

The AEs and ADRs are summarized in Table 2. A total of 102 AEs were observed in four patients from the beginning to the end and among them, AEs observed in two or more patients were upper respiratory tract inflammation, contact dermatitis, hordeolum, hypokalemia, and upper respiratory tract infection (in two patients each), and none of them were related to the study drug nor led to treatment discontinuation. A total of 21 serious adverse events (SAEs) occurred in three patients and included upper respiratory tract infection, respiratory syncytial virus infection, aspergillus infection, neutropenia, dehydration, pulmonary hemorrhage, respiratory failure, upper respiratory tract inflammation, and catheter site ulcer. Of these, pulmonary hemorrhage and respiratory failure were observed in the dead infant. No patient tested positive for anti‐elapegademase antibodies at any time point; thus, no further characterization (e.g., anti‐elapegademase IgM, neutralizing antibody, and anti‐PEG antibody) was conducted.

**Table 2:** Summary of adverse events and adverse drug reactions.

Safety analysis population, *n* = 4				
Number of subjects with AEs or ADRs		%	Number of AEs or ADRs
Adverse events (AEs)		4	100	102
Severity				
Mild		0	0	20
Moderate		4	100	82
Severe		0	0	0
Seriousness				
Not serious		1	25	81
Serious		3	75	21
Withdrawn		0	0	0
Adverse drug reactions (ADRs)		0	0	0
Severity				
Mild		0	0	0
Moderate		0	0	0
Severe		0	0	0
Seriousness				
Not serious		0	0	0
Serious		0	0	0
Withdrawn		0	0	0

*Note*: %, Incidence = (Number of subjects with AEs or ADRs/Number of subjects) × 100.

## 4 DISCUSSION

The phase 3 and postmarketing clinical study for four Japanese patients with ADA deficiency described here demonstrated that ERT with elapegademase has maintained the immune functions of patients treated with pegademase and has improved the immune functions of newly diagnosed patients by normalizing dAXP levels (≤0.02 μmol/mL in erythrocytes or whole blood) and increasing an ADA enzyme activity (≥1100 U/L in serum and ≥15 µmol/h/mL in plasma). In addition, the drug was well tolerated without notable safety concerns despite the variation of the administration dose among the patients from 0.1 to 0.4 mg/kg/week. Of particular importance was that two newly diagnosed infants were enrolled in the study. Unfortunately, one infant patient died of pneumonia caused by CMV infection in the middle of the study, but the other infant patient lived without severe infections and finished the study, which allowed us to evaluate the effect of elapegademase on the physiological dynamics of dAXP levels and ADA enzyme activity and transition of immune functions from untreated to the stable conditions.

In the clinical study, the initial dose of elapegademase for the patients who had not been treated with pegademase within 60 days before study entry was 0.1 mg/kg, and the second and third ones were 0.133 mg/kg and then increased by 0.033 mg/kg when the criteria were satisfied. On the other hand, the patients treated with pegademase received elapegademase with the enzyme activity equivalent to that of pegademase by calculating with the conversion formula at the 1st to 3rd administration and the dose was increased by 0.033 mg/kg when the criteria were satisfied. Although three patients had been treated following this regime to the end of the study, Pt‐4 who suffered from severe pneumonia caused by CMV infection at study entry was treated with 0.2 mg/kg twice a week (0.4 mg/kg/week) from the 1st administration as an urgent treatment following the regimen described elsewhere.^8^ The large amount of elapegademase in a short period of time sharply increased ADA enzyme activity without any significant safety concerns and resulted in rapid improvement of his immune functions, suggesting that high doses of elapegademase (0.2 mg/kg, twice a week) can be administrated to critically ill patients with careful attention to AEs.

Pt‐2 who had been treated with pegademase before study entry had difficulty increasing her serum ADA enzyme activity above the reference range even with pegademase‐equivalent doses of elapegademase while the trough dAXP levels in her erythrocytes were no more than 0.02 μmol/µL throughout the study. As a result, the dose of elapegademase had been increased three times by 0.033 mg/kg up to 0.3 mg/kg and maintained the stable ADA enzyme activity over 1100 U/L in serum achieving successful clinical management of the patient. Together with this, the dose of elapegademase with 0.1–0.4 mg/kg through infant to adult patients seemed to be tolerable and proper for ERT (as of December 2020).

A phase 3 clinical trial of elapegademase was conducted in the United States for six patients with ADA deficiency who had been treated with pegademase before this trial.^18^ The trial was an open‐label, uncontrolled, and one‐way crossover study consisting of the pegademase lead‐in phase (at least 3 weeks), the treatment phase (21 weeks), and the maintenance phase and enrolled six patients aged 8–37 years treated with elapegademase at 0.188–0.292 mg/kg once a week. Of the six patients, five completed the treatment phase and three received the study drug for at least 135 weeks. This study found the trough plasma ADA enzyme activity of ≥15 µmol/h/mL at almost all timepoints and maintained metabolic detoxification for at least 2 years under the treatment with elapegademase. Of importance was that three patients in the United States phase 3 study showed higher trough levels of the plasma ADA enzyme activity and a 1.5‐ to 3‐fold increase in lymphocyte and its subset counts in the treatment phase compared with the lead‐in phase even though they received elapegademase equivalent to pegademase in ADA enzyme activity, and the observed increases in the enzyme activity and cell counts lasted for at least 135 weeks. In addition, a recent study compared the immune functions of an infant treated with elapegademase to those of three aged‐matched ones treated with pegademase and showed a comparable increase of T cell counts after 3–4 months of treatment against the controls.^19^

AEs observed in this study were tolerable and acceptable and none of them were of particular concern. The US study also showed no severe AEs and common ones, if any, such as cough (3 pts) and vomiting (2 pts) regardless of causality,^18^ although the future direction would be to collect data on the long‐term safety due to very limited information available. In terms of the production of an antibody against pegademase including anti‐drug or anti‐polyethylene glycol causative for an unexplained decrease in ADA activity,^18^ no antibody against elapegademase was detected in this study. One plausible explanation is that elapegademase is a genetically modified product unlike pegademase, which is a biological one derived from bovine serum ADA.

The study was designed as an open‐label, uncontrolled study because ADA deficiency is a severe inherited disorder without any effective treatments except for HCT from HLA‐identical sibling donors in Japan and the use of a placebo control was not acceptable ethically. Despite these limitations, this study together with the US study has provided data on the safety and efficacy of the ERT with elapegademase for patients with ADA deficiency over 150 weeks. However, it is true that they still need prophylactic antibiotics and gamma globulin replacement therapy. In addition, long‐term ERT has been reported to compromise patient immunity. Thus, we believe that patients should undergo gene therapy if HSC‐GT becomes available in Japan. On the other hand, in situations where gene therapy is not available and patient immunity cannot be maintained, HCT from a suitable donor should be considered without delay. Careful long‐term follow‐up of patients should be continued.

In conclusion, this study has revealed that elapegademase is safe, maintains sufficient ADA activity, and reduces toxic metabolites. On the other hand, although the patients' immune functions improved to live without serious infections, it is necessary to continue to carefully evaluate immune function during long‐term follow‐up since the observation period was quite short.

## AUTHOR CONTRIBUTIONS

*Conception and design*: Masafumi Onodera, Tadashi Ariga, Hironori Arizono, and Tomohiro Morio. *Acquisition of data*: Masafumi Onodera, Toru Uchiyama, Tadashi Ariga, Masafumi Yamada, and Takako Miyamura. *Analysis and interpretation of data*: Masafumi Onodera, Hironori Arizono, and Tomohiro Morio. *Drafting the manuscript*: Masafumi Onodera. *Revised the manuscript*: All authors. All authors consented to the publication of the final version of the manuscript.

## CONFLICTS OF INTEREST STATEMENT

Masafumi Onodera received fees from Teijin Pharma Limited for his consultations on this study. Tadashi Ariga received fees from Teijin Pharma Limited for his consultations of this study (including travel expenses for consultations). Tomohiro Morio received fees from Teijin Pharma Limited for his consultations on this study. Hironori Arizono is an employee of Teijin Pharma Limited. The remaining authors declare no conflict of interest.

## Supporting information

Supporting information.

## Data Availability

Research data are not shared.
