# Supplementary material for: Safety and efficacy of elapegademase in patients with adenosine deaminase deficiency: A multicenter, open‐label, single‐arm, phase 3, and postmarketing clinical study
Source: Immun Inflamm Dis. 2023 Jul 25;11(7):e917. doi: 10.1002/iid3.917 (PMC10367445; doi:10.1002/iid3.917)
Supplement: Supplementary file 1 — Supporting information. [file IID3-11-e917-s001.docx]

Supplementary Table. Dosage and administration records

| Patient code, dosage and administration | | | Patient-1,  once a week, mg/kg | Patient-2,  once a week, mg/kg | Patient-3,  once a week, mg/kg | Patient-4,  twice a week, mg/kg |
| --- | --- | --- | --- | --- | --- | --- |
| Evaluation phase | | |  |  |  |  |
|  | Dose adjustment period | |  |  |  |  |
|  |  | 1st administration | 0.1 | 0.2 | 0.167 | 0.2 x 2 |
|  |  | 2nd | 0.133 | 0.2 | 0.167 | 0.2 x 2 |
|  |  | 3rd | 0.133 | 0.2 | 0.167 | 0.2 x 2 |
|  |  | 4th | 0.167 | 0.2 | 0.2 | 0.2 x 2 |
|  |  | 5th | 0.167 | 0.2 | 0.2 | 0.2 x 2 |
|  | Dose maintenance period | |  |  |  |  |
|  |  | 6th | 0.167 | 0.2 | 0.2 | 0.2 x 2 |
|  |  | 7th | 0.167 | 0.2 | 0.2 | 0.2 x 2 |
|  |  | 8th | 0.167 | 0.2 | 0.2 | 0.2 x 2 |
|  |  | 9th | 0.167 | 0.2 | 0.2 | 0.2 x 2 |
|  |  | 10th | 0.167 | 0.233 | 0.233 | 0.2 x 2 |
|  |  | 11th | 0.167 | 0.233 | 0.233 | 0.2 x 2 |
|  |  | 12th | 0.167 | 0.233 | 0.233 | 0.2 x 2 |
|  |  | 13th | 0.167 | 0.233 | 0.233 | 0.2 x 2 |
|  |  | 14th | 0.167 | 0.233 | 0.233 | 0.2 x 2 |
|  |  | 15th | 0.167 | 0.233 | 0.233 | 0.2 x 2 |
|  |  | 16th | 0.167 | 0.233 | 0.233 | 0.2 |
|  |  | 17th | 0.167 | 0.233 | 0.233 | ― |
|  |  | 18th | 0.167 | 0.267 | 0.233 | ― |
|  |  | 19th | 0.167 | 0.267 | 0.233 | ― |
|  |  | 20th | 0.167 | 0.267 | 0.233 | ― |
|  |  | 21st | 0.167 | 0.267 | 0.233 | ― |
| Continuous administration phase | | |  |  |  |  |
|  |  | 22nd | 0.167 | 0.267 | 0.233 | ― |
|  |  | 38th | 0.167 | 0.3 | 0.233 | ― |
|  |  | 164th | 0.167 | 0.3 | 0.233 | ― |
|  |  | 165th | ― | 0.3 | 0.233 | ― |
|  |  | 169th | ― | 0.3 | ― | ― |
